# Surgical treatment of liver metastases from non-colorectal non-neuroendocrine carcinomas

**DOI:** 10.1007/s00432-021-03631-5

**Published:** 2021-04-20

**Authors:** Astrid Bauschke, Annelore Altendorf-Hofmann, Merten Homman, Thomas Manger, Jörg Pertschy, Herry Helfritzsch, Hubert Göbel, Utz Settmacher

**Affiliations:** 1 Department of General, Visceral and Vascular Surgery, University Hospital Jena, Erlanger Allee 101, 07740 Jena, Germany; 2 Department of General, Visceral Surgery, Zentralklinik Bad Berka, Robert-Koch-Allee 9, 99438 Bad Berka, Germany; 3 Department of General, Visceral Surgery, SRH Wald-Klinikum Gera GmbH, Str. des Friedens 122, 07548 Gera, Germany; 4 Department of General, Visceral and Vascular Surgery, Katholisches Krankenhaus Erfurt, Haarbergstraße 72, 99097 Erfurt, Germany; 5 Thüringen-Kliniken “Georgius Agricola, Rainweg 68, 07318 Saalfeld, Germany; 6 Clinical Cancer Registry Thuringia with Tumor Center e.V. Erfurt HELIOS Klinikum Erfurt GmbH, Haus 22, Nordhäuser Str. 74, 99089 Erfurt, Germany

**Keywords:** Non-colorectal non-neuroendocrine liver metastases, Surgery, Survival

## Abstract

**Introduction:**

In the literature, results after surgical treatment of non-colorectal non-neuroendocrine liver metastases (NCNNLM) are reported that are often inferior to those from colorectal liver metastases. The selection of patients with favorable tumor biology is currently still a matter of discussion.

**Materials/methods:**

The retrospective data analysis was based on data that were collected for the multicenter study “Role of surgical treatment for non-colorectal liver metastases” in county Thuringia.

**Results:**

For the study, 637 patients were included from 1995 to 2018. 5 and 10-year survival of R0 resected patients were 33% and 19%, respectively. In the multi-variate analysis of the entire group, sex, timing, disease-free interval, number of metastases, R-classification as well as lymph node status of the primary lesion showed an independent statistical influence on the 5-year survival. In the group of R0 resected patients, disease-free interval, number of metastases and lymph node status of the primary lesion influenced the 5-year survival in the multi-variate analysis. In kidney malignancies, R-classification, timing and number of liver metastases were statistically significant in the multi-variate analysis of the 5-year survival, in mamma carcinomas only the R-classification.

**Conclusion:**

The Adam score identifies some risk factors which influence prognosis in most but not in all tumor entities. For kidney cancer and breast cancer it can be simplified.

## Introduction

The effect of surgical therapy of non-colorectal non-neuroendocrine carcinomas is still under debate. Due to small incidence of liver metastases suitable for complete resection, only a few studies can give guidelines or recommendations for systemic or local therapy. Even in nationwide studies (Grimme et al. 2019; Ruys et al. 2011), the number of included patients remains small. Other than in colorectal carcinoma, in many solid carcinoma liver metastases are a predictor of more widespread disease. For many solid types of cancer effective hormone-, chemo- and immune-therapies exist. Often patients are presented to the surgeon when systemic therapies lead to progressive disease.

Most studies summarize a broad variety of cancer entities with differing prognosis to groups. Only for breast cancer (Feng et al. 2020), kidney cancer (Bauschke et al. 2021; Ruys et al. 2011), gastric cancer (Luo et al. 2019), and sarcoma (Grimme et al. 2019) exist a small number of studies with adequate sample size to help clinicians to make a decision for or against surgical therapy.

We present data from patients treated surgically in a German federal state.

## Materials/methods

This retrospective data analysis is based on the data of the multi-center study “Role of surgical treatment of non-colorectal liver metastases in Thuringia”. The study in human subjects was carried out with consent of the local ethics committee (ethical vote 5073-02/17) in accordance with national law and the Declaration of Helsinki of 1975 (in the current revised form).

From the five tumor centers in the country of Thuringia, we requested a list of all patients with OP codes 5-501.*, 5-502.* or 5-504.* (liver resection or liver transplantation, respectively) (Fritz 2013) in malignant primary tumors, except colorectal primaries, primary liver cancer, hilar cholangiocarcinoma and systemic diseases between 1995 and 2018. Data not found in the cancer registries of the five tumor centers were completed by contacting clinicians.

The participating hospitals were in addition to one university hospital, seven maximum care facilities and 12 other hospitals. Only patients with histologically confirmed liver metastases were included. Primary liver tumors, benign liver tumors, patients with direct invasion of the liver by peritoneal implants or by the primary tumor were excluded. Patients underwent routine staging using preoperative computed tomography scanning of the chest, abdomen, and pelvis.

Morphology was classified according to the manual of cancer registration (Stegmaier et al. 2019). In case of bilobar metastases smaller than 5 cm, radiofrequency ablation was performed alone or in combination with liver resection. In case of combined resection and radiofrequency ablation, the procedure was classified as radiofrequency ablation. If all tumor locations had been successfully treated with radiofrequency ablation, it was classified as R0, in case of further remnant tumor locations as R2 situation.

The aim of surgery was always complete removal of all present tumor (R0 resection). Surgical options have been limited by intraoperative non-resectable primary tumors, but also by unknown diffuse liver metastases or non-resectable other tumor locations. In individual cases, regional lymphadenectomy was performed.

### Statistical methods

All statistical analyses were performed using SPSS 26.0 (IBM, Chicago, IL, USA) software. Categorical variables were tested for independence using the Chi-squared test or Fisher’s exact test as indicated. Survival was calculated from the date of liver resection. Overall survival (patients’ death irrespective of the cause of death) was used as the endpoint for estimating prognosis. The median follow-up time was calculated using the reverse Kaplan–Meier method. Survival curves were created using the Kaplan–Meier method, and the log-rank test was used to assess differences in survival. Significant and independent predictors of overall survival were identified by Cox proportional hazard analysis. The procedure was set to a threshold of 0.05. Statistical significance was defined as a *p* value < 0.05 for all analyses.

## Results

This study analyzed 637 patients who underwent treatment in 20 Thuringian hospitals from 1995 to 2018. We analyzed location, morphology, and additionally 11 patient characteristics, also metastases, primary tumor lesion, liver metastases, surgical procedure, and individual hospital experience. 86% of the patients (546) underwent treatment in the nine transregional hospitals, 14% (91) in regional hospitals. 15 hospitals treated less than 40 patients, four hospitals between 40 and 100 patients, and the university hospital treated 213 patients.

In the hospitals that treated < 40 patients, there were statistically significantly more less-than-radical procedures, limited resections, metastases following tumor free interval < 24 months and non-resectable primary tumors (*p* < 0.001 each).

Half of the 637 patients had extrahepatic tumor at the time of surgery. Only in 38% of the cases complete resection was accomplished. Details are shown in Table 1.

**Table 1:** Patients under study

Category	Prognostic factor	Strata	Patients	%
Patient	Age	< 60	253	40
		≥ 60	384	60
	Sex	Male	303	48
		Female	334	52
Hepatic metastases	Timing	Metachronous	355	56
		Synchronous	282	44
	Disease-free interval	< 12 months	345	54
		12–24 months	71	11
		> 24 months	221	35
	Number	Solitary	262	41
		Multiple	375	59
	Size	< 5 cm	482	76
		> 5 cm	155	24
	Extrahepatic disease	Present	320	50
		Absent	317	50
	Adam risk score	Low	136	22
		Intermediate	372	58
		High	129	20
Hepatectomy	Extent of liver resection	Limited	494	78
		Major	143	22
	Margin of liver resection	R0	280	44
		R1	24	4
		R2	333	52
Primary tumor	N-category of primary tumor	N−	289	45
		N +	210	33
		Not removed	138	22
Hospital	Number of patients	University	213	33
		≥ 40	249	39
		< 40	175	28

6 of the 26 different primary tumors (lung, ovary, kidney, stomach, breast, pancreas) are assigned to 86% of patients. In these, the proportion of R0 resections ranged between 16% (pancreas) and 64% (lung).

In 75 patients (12%), radiofrequency ablation of metastases was performed, sometimes in combination with liver resection. Complete macroscopic tumor resection was accomplished in 39 patients. In a total of 280 (44%) patients, an R0 situation was thus achieved, in 24 (4%) an R1 resection was performed. The primary tumor was non-resectable in 138 (22%) patients, in the remaining 195 (31%) patients, metastases were non-resectable (Table 2).

**Table 2:** R-classification according to location of the primary tumor

Location	Patients	R0		R1		R2		Thermo-ablation, R0		Thermo-ablation, R2	
Esophagus	17	6	35%	0	0%	9	53%	0	0%	2	12%
Stomach	94	44	47%	4	4%	33	35%	10	11%	3	3%
Small intestine	19	10	53%	0	0%	8	42%	0	0%	1	5%
Anus, anal canal	5	4	80%	0	0%	1	20%	0	0%	0	0%
Pancreas	143	23	16%	3	2%	110	77%	5	3%	2	1%
Ear, nose, throat area	5	3	60%	0	0%	1	20%	0	0%	1	20%
Lung	22	14	64%	0	0%	5	23%	1	5%	2	9%
Thymus	1	0	0%	0	0%	1	100%	0	0%	0	0%
Bones	1	0	0%	0	0%	1	100%	0	0%	0	0%
Skin	11	5	45%	0	0%	4	36%	1	9%	1	9%
Retroperitoneum	7	2	29%	0	0%	4	57%	1	14%	0	0%
Peripheral soft tissue	2	1	50%	0	0%	1	50%	0	0%	0	0%
Mamma	117	46	39%	4	3%	37	32%	14	12%	16	14%
Cervix/uterus	19	9	47%	2	11%	7	37%	0	0%	1	5%
Gall bladder/bile ducts	5	2	40%	1	20%	1	20%	1	20%	0	0%
Ovary	38	11	29%	4	11%	18	47%	1	3%	4	11%
Prostate	8	1	13%	1	13%	5	63%	0	0%	1	13%
Testes	4	1	25%	0	0%	3	75%	0	0%	0	0%
Kidney	70	40	57%	4	6%	21	30%	3	4%	2	3%
Renal pelvis	4	2	50%	0	0%	2	50%	0	0%	0	0%
Ureter	1	0	0%	0	0%	1	100%	0	0%	0	0%
Urinary bladder	6	3	50%	1	17%	2	33%	0	0%	0	0%
Eye	18	9	50%	0	0%	7	39%	2	11%	0	0%
Thyroid gland	4	2	50%	0	0%	2	50%	0	0%	0	0%
Adrenal gland	4	3	75%	0	0%	1	25%	0	0%	0	0%
Unknown primary tumor	12	0	0%	0	0%	12	100%	0	0%	0	0%
Total	637	241	38%	24	4%	297	47%	39	6%	36	6%

Most frequently (78%), the diagnosis was adenocarcinoma, followed by squamous cell carcinoma (7%), sarcomas and melanomas (5% each), gastrointestinal stromal tumors (GIST) (2%), and 3% other tumors (three patients each with malignant granulosa cell tumor, unclassified carcinoma, two patients each with malignant mixed mullerian tumor, papillary carcinoma, pseudo sarcomatous carcinoma, undifferentiated carcinoma and one patient each with peripheral neuro-ectodermal tumor, choroid carcinoma, mixed germ-cell carcinoma, yolk sac carcinoma, embryonic carcinoma, adeno-squamous carcinoma or anaplastic carcinoma.

### Morbidity/30-day mortality

Among the 346 limited procedures, there were 254 atypical resections and 66 radiofrequency ablations. Among atypical resections, one hemorrhage and one liver abscess were documented. Another liver abscess required treatment after radiofrequency ablation. In the remaining 289 cases, 60 complications were recorded resulting in 21% morbidity (Clavien II–V). Seven of these patients died post-operatively, four after R0, and three after R2 resection.

### Long-term survival

The median follow-up period in all patients was 107 months.

The 5- and 10-year survival rates in all patients were 18% and 9%, respectively, median survival time was 16 months. So far, 86 patients have survived liver surgery longer than five years and 26 patients have survived the procedure for longer than 10 years. Primaries were nine breast cancers, four GISTs, four kidney tumors, two adenocarcinomas of stomach or ovary, one squamous cell carcinoma of each lung, esophagus, larynx, as well as one malignant melanoma of the skin, and one leiomyosarcoma of the small intestine.

If all detectable tumor was removed by radiofrequency ablation, the 5-year survival rate was not significantly different from that after R0 resection. After R2 procedures, the survival rates were almost identical for resection and radiofrequency ablation.

The survival rates in all 637 cases have been pair-wise statistically significantly different, depending on the number of patients treated per hospital. If one considers only the 241 R0 resected patients, survival rates are almost identical.

With respect to the individual locations of the primary tumor, the 5-year survival rate was between 0% (pancreas) and 30% (kidney) (Table 3).

**Table 3:** 5-, 10-year survival rates according to location and morphology of the primary tumor

Study population	Patients	%	5-year survival (%)	10-year survival (%)	Median survival (months)
All patients	637	100	18	9	16
Location					
Pancreas	143	22	0	0	8
Lung	22	4	8	8	19
Other PT^a^	87	14	13	6	18
Esophagus/stomach/small intestine	130	20	20	11	16
Female genitals	57	9	23	9	27
Mamma	117	18	29	18	31
Kidney/renal pelvis	82	13	30	10	23
Morphology					
Squamous cell	43	7	8	8	8
Melanoma	29	5	11	11	16
Adenocarcinoma	499	78	16	8	15
Other morphologie	21	3	18	9	12
Sarcoma	29	5	30	14	36
Stromal GIST	16	2	75	39	104

^a^Other PT primary tumor: unknown primary tumor—12, adrenal gland—4, thyroid gland—4, eye—18, testes—4, prostate—8, gall bladder/bile ducts—5, peripheral soft tissue—2, retroperitoneum—7, skin—11, bones—1, thymus—1, ear nose and throat area—5, anus and anal canal—5

**Fig. 1:**
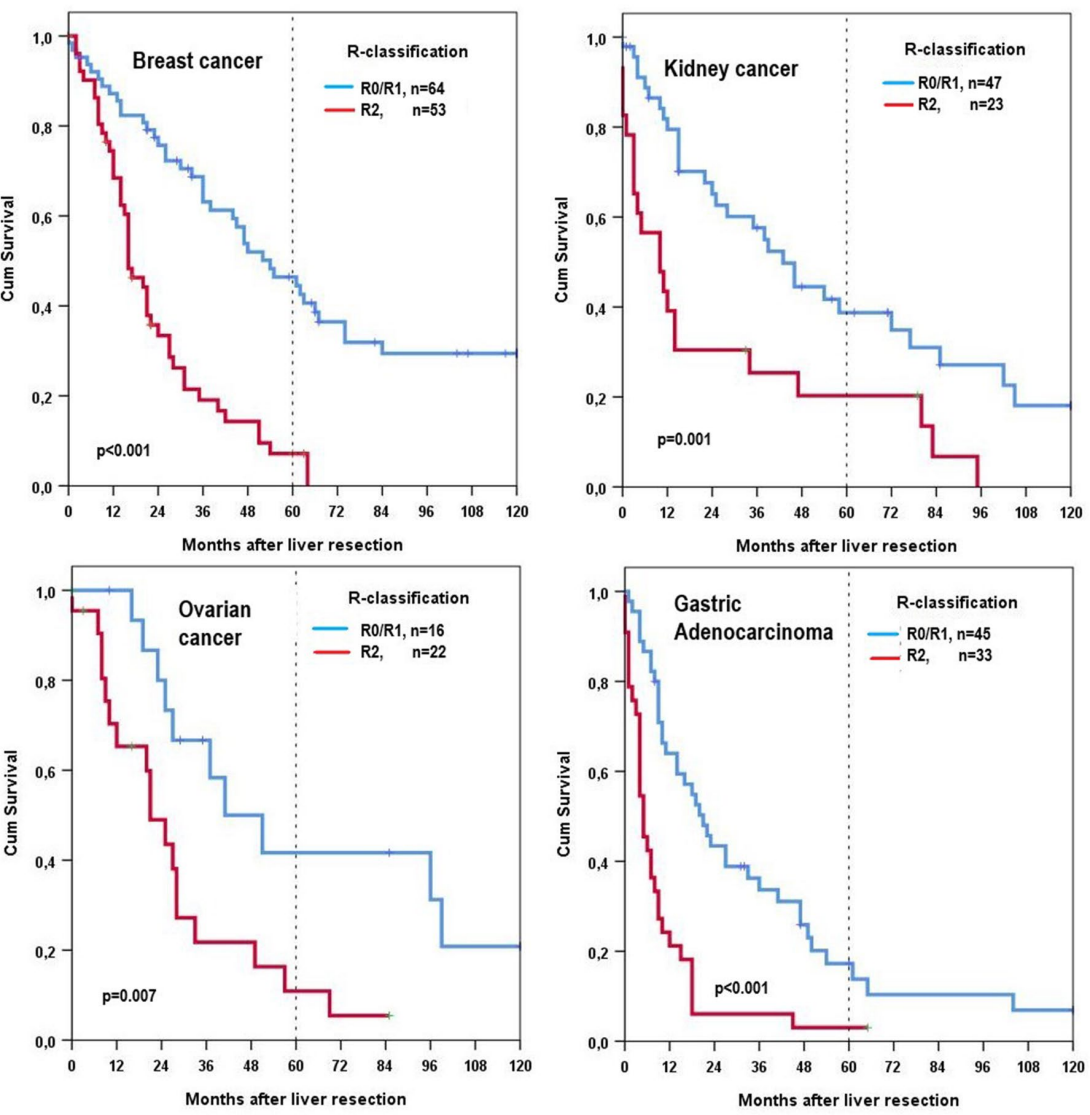
Overall survival of patients with breast cancer, kidney cancer, ovarian cancer, and adenocarcinoma of the stomach

For 64 radically resected patients (R0/R1) and patients with breast carcinoma, the 5- and 10-year overall survival rates were 46% and 29%, respectively, for ovarian cancer liver metastases 41% and 21%, respectively, for kidney cancer liver metastases 39% and 18%, and for gastric adenocarcinoma 17% and 7%, respectively (Fig. 1). None of our patients with pancreatic adenocarcinoma survived 5 years, the median survival time for the 23 R0-resected patients was 23 months.

**Fig. 2:**
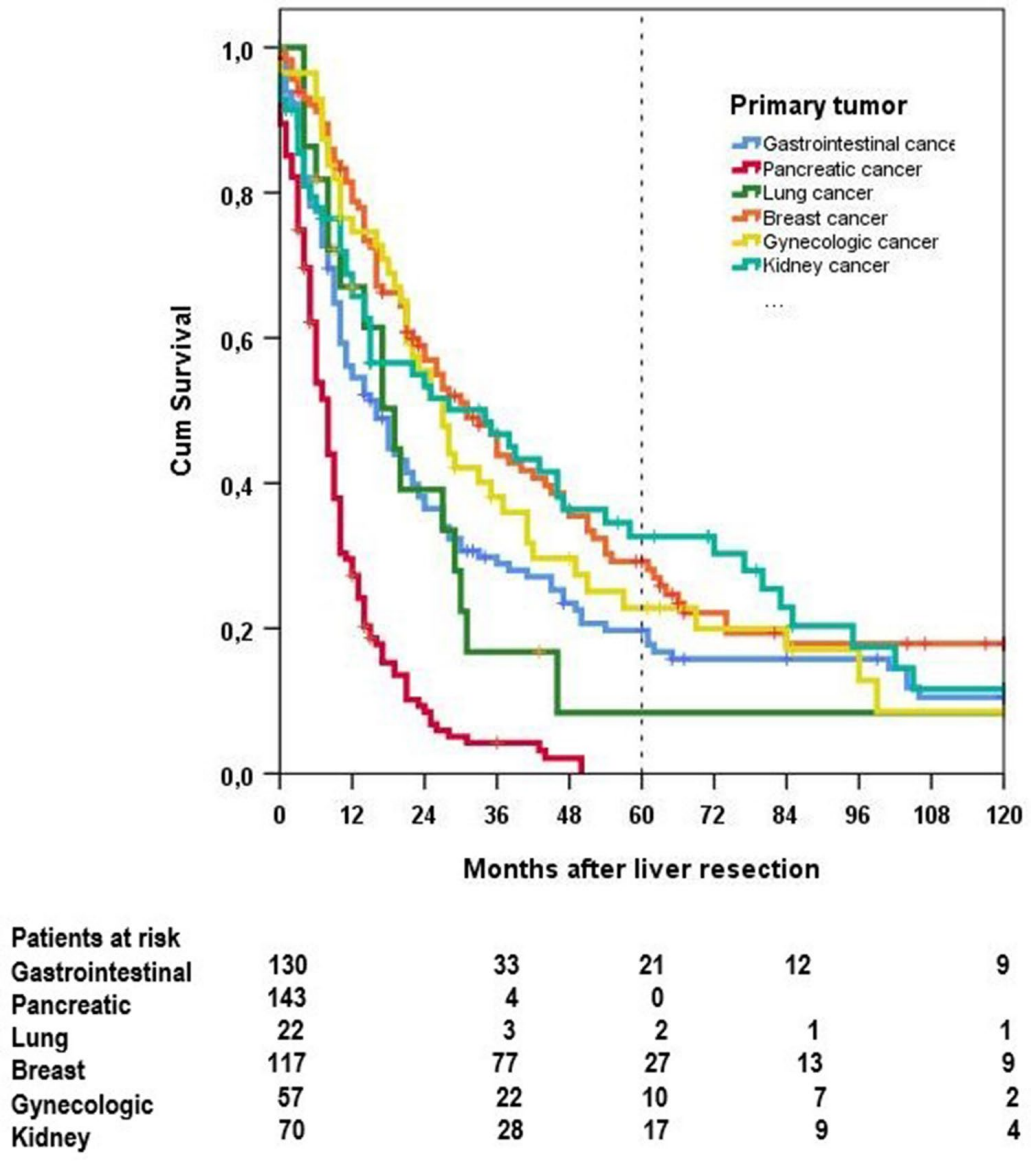
Overall survival dependent on location of the primary

Patients with liver metastases from breast cancer had a better 5- and 10-year survival than from lung malignancies and gastrointestinal tumors (*p* = 0.005). Patients with liver metastases from adenocarcinomas of the pancreas had a significantly poorer survival than all other malignancies (Fig. 2). Liver metastases from breast cancer had a better survival than from gastrointestinal tumors and lung malignancies (*p* = 0.026) (Table 3). For breast cancer, one needs to consider that immunotherapy and chemotherapy may influence the prognosis in the metastatic stage markedly.

Still, patients with breast cancer are investigated separately by some authors, as with the introduction of immunotherapy and chemotherapy the prognosis was markedly improved.

Location only does not always provide for homogenous groups. For breast and kidney, only metastases from adenocarcinomas were observed. In the esophagus, there were half adenocarcinomas or squamous cell carcinomas each, respectively; in the stomach in addition to adenocarcinomas also 11% GIST and 2% sarcomas.

**Fig. 3:**
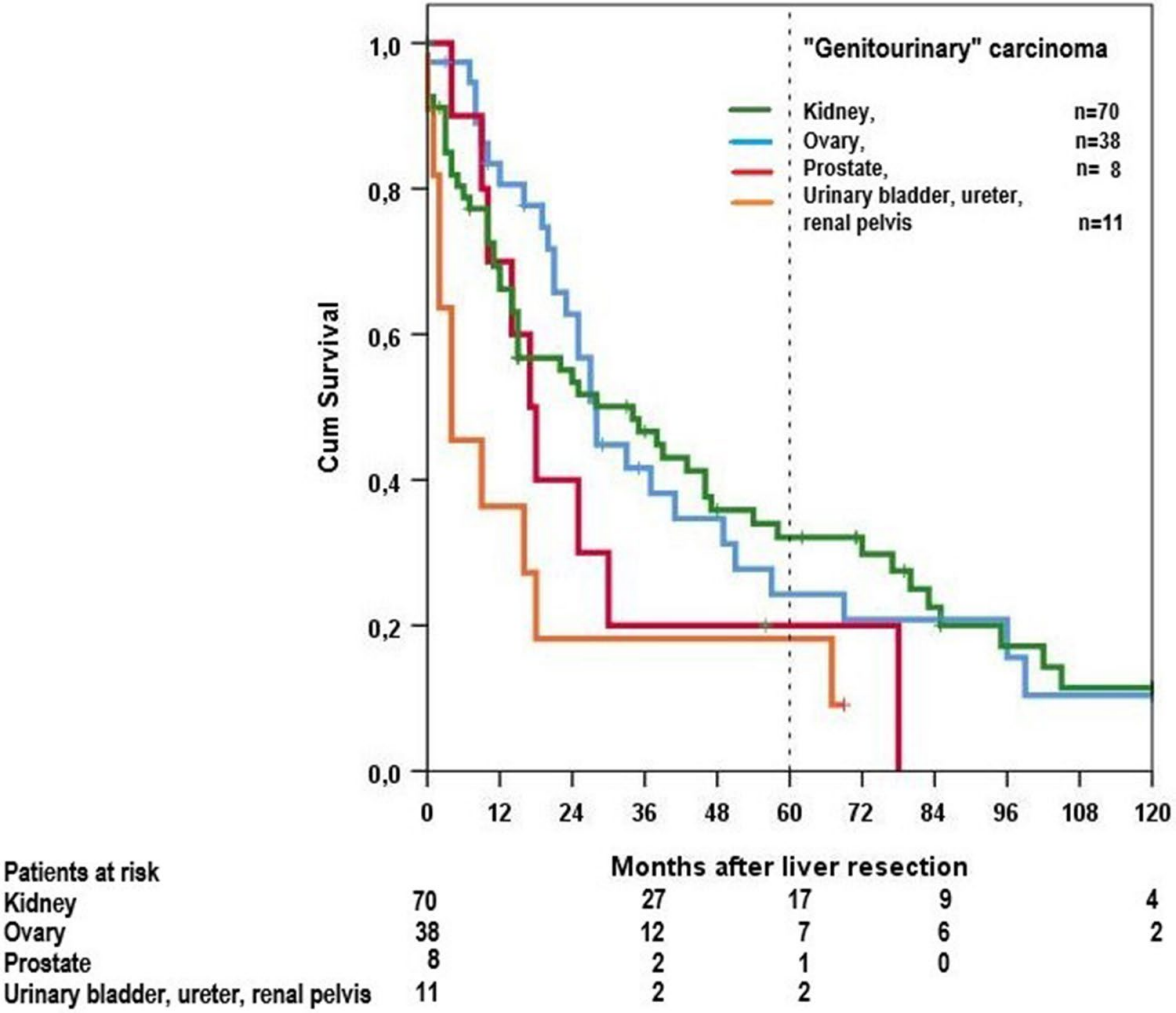
Overall survival of patients with genitourinary cancer

In our study, there is a statistically significant difference in survival between urinary bladder, ureter, and renal pelvis versus ovary (*p* = 0.012) and versus kidney (*p* = 0.017) (Fig. 3).

**Fig. 4:**
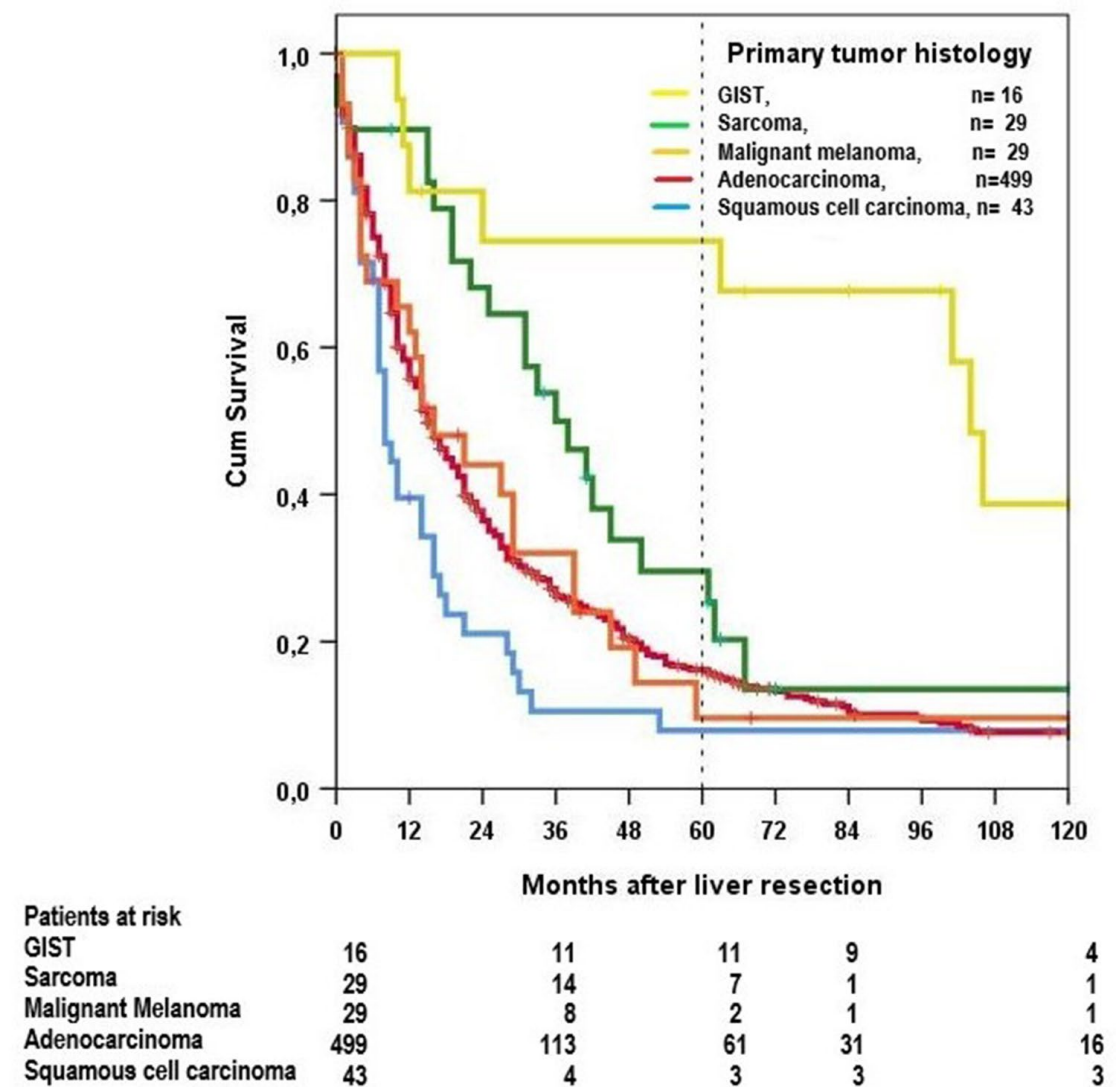
Overall survival dependent on histology

**Fig. 5:**
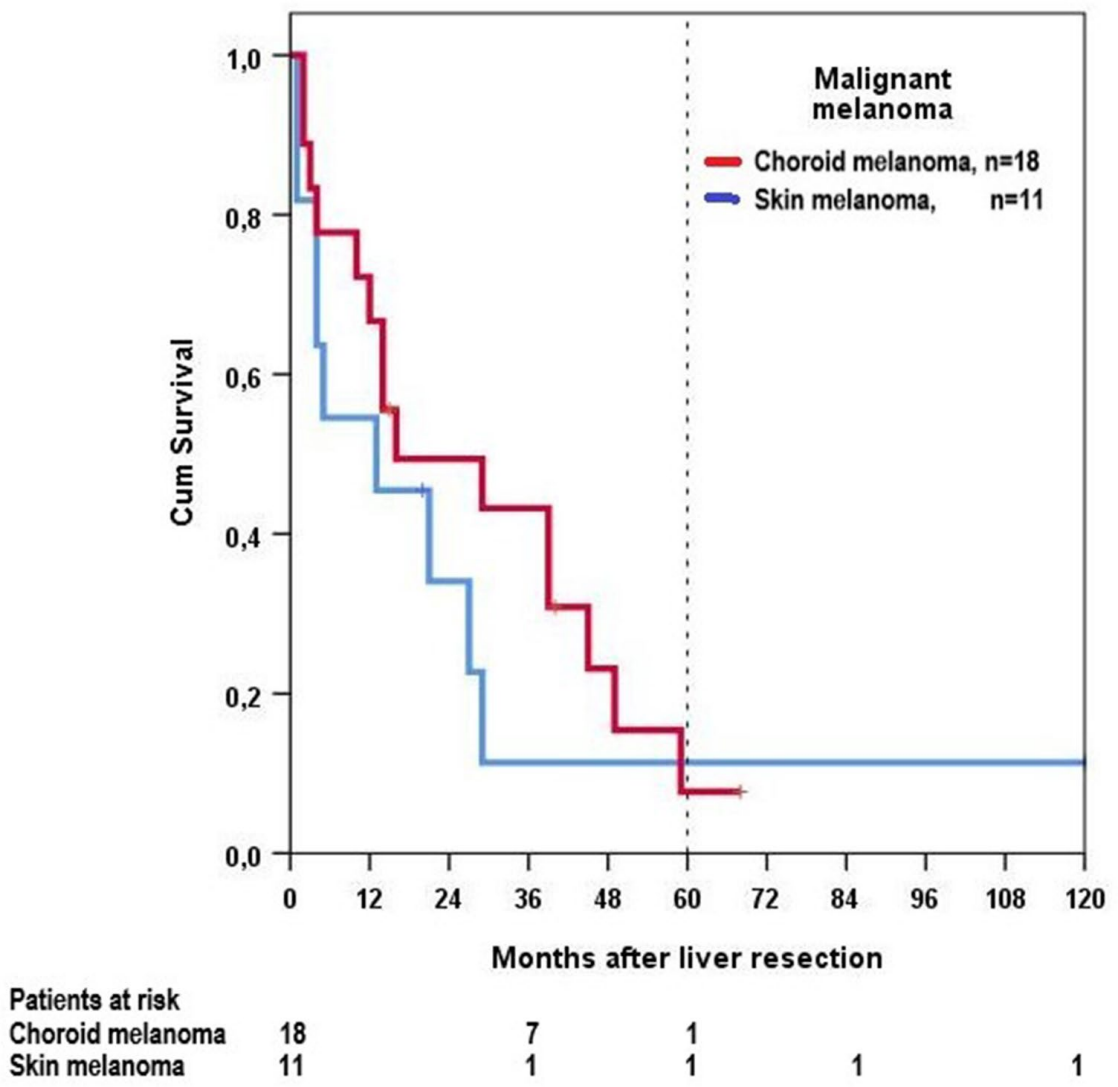
Overall survival of patients with malignant melanoma

The highest 5-year survival rates (75%) were achieved in GIST, the lowest in squamous cell carcinoma (8%). Long-term survival of the 16 GISTs was statistically significantly better than that of all other groups. Sarcomas had a statistically significantly better long-term survival than squamous cell carcinomas and adenocarcinomas. The difference between squamous cell carcinomas and sarcomas or adenocarcinomas, too, was statistically significant, but not the difference between malignant melanomas and adenocarcinomas or squamous cell carcinomas (Table 3, Fig. 4). In malignant melanomas there was no statistically significant difference in long-term survival between 18 choroid melanomas and 11 skin melanomas (Fig. 5).

While in the univariate analysis of all 637 patients, all investigated factors had a statistically significant impact on the 5-year survival; in the multivariate analysis only sex, timing, disease-free interval, number of metastases, R-classification, lymph node status of the primary lesion as well as remaining primary lesion had an independent statistically significant impact on the 5-year survival. In the 241 R0 resected patients, disease-free interval, number of metastases, and lymph node status of the primary lesion showed an independent statistically different impact on survival in the multivariate analysis (Table 4).

**Table 4:** Uni- and multi-variate analyses of all patients and of the R0 resected

All patients, *n* = 637		Univariate		Multivariate	
Prognostic factor	Strata	Significance	Exp (B) (95% CI)	Significance	Exp (B) (95% CI)
Age		**0.007**	1.012 (1.003–1.021)	0.108	1.007 (0.998–1.016)
Sex	Female/male	**0.000**	1.615 (1.349–1.933)	**0.005**	1.315 (1.087–1.591)
Timing	Metachronous/synchronous	**0.000**	2.331 (1.942–2.798)	**0.006**	1.418 (1.104–1.822)
Disease-free interval	> 24 months/0–24 months	**0.000**	1.990 (1.647–2.404)	**0.047**	1.304 (1.004–1.695)
Number of liver metastases	Solitary/multiple	**0.000**	1.990 (1.647–2.404)	**0.000**	1.525 (1.233–1.887)
Size of lesion	≤ 5 cm/> 5 cm	**0.008**	0.750 (0.607–0.926)	0.967	0.995 (0.779–1.270)
Extrahepatic disease	Absent/present	**0.000**	0.606 (0.506–0.726)	0.651	1.056 (0.833–1.340)
Extent of liver resection	Limited/major	**0.000**	1.514 (1.215–1.887)	0.819	1.031 (0.793–1.341)
Margin of liver resection	R0/R1-2	**0.000**	2.436 (2.002–2.965)	**0.000**	1.829 (1.415–2.364)
N-category of primary tumor	N0/N+/primary tumor not removed			**0.011**	
		**0.001**	1.427 (1.161–1.755)	**0.023**	1.283 (1.035–1.589)
		**0.000**	3.379 (2.666–4.282)	**0.009**	1.497 (1.104–2.031)
Number of patients per hospital	University/> 40 patients/ ≤ 40 patients			0.530	
		**0.002**	1.396 (1.125–1.733)	0.268	1.151 (0.898–1.475)
		**0.000**	1.777 (1.415–2.233)	0.386	1.130 (0.857–1.489)
R0, *n* = 241					
Sex	Female/male	**0.003**	1.618 (1.172–2.233)	0.056	1.385 (0.991–1.935)
Timing	Metachronous/synchronous	**0.000**	2.251 (1.618–3.132)	0.083	1.424 (0.955–2.125)
Disease-free interval	> 24 months/0–24 months	**0.000**	2.373 (1.668–3.375)	**0.015**	1.692 (1.108–2.583)
Number of liver metastases	Solitary/multiple	**0.019**	1.490 (1.069–2.077)	**0.016**	1.517 (1.082–2.127)
N-category of primary tumor	N0/N+	**0.001**	1.702 (1.230–2.354)	**0.020**	1.489 (1066–2.081)

Bold indicates *p* < 0.005

We further investigated the prognostic factors for long-term survival in adenocarcinomas, gastrointestinal carcinomas, breast cancers and kidney carcinomas. The respective statistically significant prognostic factors were included in the multivariate analysis.

In the multivariate analysis of all 499 adenocarcinomas, sex, timing, disease-free interval, number of metastases, and R-classification had a statistically significant impact. Analysis of the 94 gastrointestinal adenocarcinomas showed that only age had a statistically significant impact (*p* = 0.036) on the observed 5-year survival (Table 5). In liver metastases from breast cancer only the R-classification had an independent statistically significant impact on the 5-year survival. In kidney carcinomas, the number of liver metastases, the interval between resection of the primary lesion and the diagnosis of liver metastases as well as the R-classification had an independent statistically significant impact on the 5-year survival (Table 6).

**Table 5:** Uni- and multi-variate analyses of patients with adenocarcinomas or gastrointestinal adenocarcinomas

Adenocarcinoma, *n* = 499		Univariate		Multivariate	
Prognostic factor	Strata	Significance	Exp (B) (95% CI)	Significance	Exp (B) (95% CI)
Age		**0.001**	1.016 (1.007–1.027)	0.078	1.009 (0.999–1.020)
Sex	Female/male	**0.000**	1.599 (1.307–1.957)	**0.022**	1.281 (0.036–1.584)
Timing	Metachronous/synchronous	**0.000**	2.576 (2.095–3.167)	**0.019**	1.433 (1.061–1.934)
Disease-free interval	> 24 months/0–24 months	**0.000**	2.427 (1.930–3.053)	**0.004**	0.640 (0.471–0.870)
Number of liver metastases	Solitary/multiple	**0.000**	2.215 (1.787–2.745)	**0.000**	1.614 (1.265–2.060)
Size of lesion	≤ 5 cm/> 5 cm	**0.001**	0.654 (0.510–0.841)	0.356	0.873 (0.655–1.165)
Extrahepatic disease	Absent/present	**0.000**	0.585 (0.478–0.716)	0.911	1.015 (0.776–1.329)
Extent of liver resection	Limited/major	**0.000**	1.640 (1.265–2.128)	0.955	1.009 (0.738–1.379)
Margin of liver resection	R0/R1-2	**0.000**	2.451 (1.963–3.059)	**0.000**	1.814 (1.359–2.421)
N-category of primary tumor	N0/N+/primary tumor not removed	**0.000**		0.265	
		0.073	1.239 (0.980–1.565)	0.623	1.063 (0.833–1.356)
		**0.000**	3.517 (2.696–4.587)	0.103	1.334 (0.943–1.887)
Number of patients per hospital	University/> 40 patients/≤ 40 patients	**0.000**		0.732	
		**0.005**	1.416 (1.108–1.809)	0.491	1.104 (0.834–1.460)
		**0.000**	1.810 (1.396–2.347)	0.457	1.127 (0.823–1.543)

Bold indicates *p* < 0.005

**Table 6:** Uni- and multi-variate analyses of patients with of breast or kidney carcinomas

Prognostic factor	Strata	Univariate		Multivariate	
		significance	Exp (B) (95% CI)	significance	Exp (B) (95% CI)
Breast, *n* = 117					
Number of liver metastases	Solitary/multiple	**0.001**	2.253 (1.409–3.603)	0.060	1.617 (0.980–2.668)
Size of lesion	≤ 5 cm / > 5 cm	**0.076**	1.547 (0.955–2.506)	0.267	1.321 (0.808–2.158)
Extrahepatic disease	Absent/present	**0.001**	0.457 (0.287–0.726)	0.050	0.609 (0.370–1.000)
Margin of liver resection	R0/R1-2	**0.000**	3.191 (1.883–5.408)	**0.014**	2.140 (1.169–3.918)
Kidney, *n* = 70					
Sex	Female/male	**0.186**	1.590 (0.800–3.161)	0.371	1.404 (0.668–2.951)
Timing	Metachronous/synchronous	**0.016**	2492 (1.185–5.243)	**0.014**	3.545 (1.286–9.771)
Disease-free interval	> 24 months/0–24 months	**0.003**	2.531 (1.383–4.631)	0.499	0.768 (0.358–1.650)
Number of liver metastases	Solitary/multiple	**0.004**	2.588 (1.343–4.985)	**0.015**	2.597 (1.208–5.584)
Margin of liver resection	R0/R1-2	**0.004**	2.430 (1.325–4.455)	**0.035**	2.045 (1.051–3.979)

Bold indicates *p* < 0.005

## Discussion

Non-colorectal non-neuroendocrine liver metastases are far from being a homogenous group. A resilient search for prognostic factors requires a minimum number of patients. Thus, most studies are published from highly specialized hospitals or a group of them. In the last years, liver surgery, limited resections in particular, can be performed with low morbidity and mortality (Table 7). In consequence, smaller hospitals, too, perform liver resections. Yet, surgical decision making in rare entities, such as non-colorectal liver metastases is extremely complex while guidelines for these are lacking.

**Table 7:** Studies > 100 patients with non-colorectal non-neuroendocrine liver metastasis since 2005

	Period	Number of patients	GIST %	Melanoma %	Sarcoma %	Morbidity (> Clavien 3) %	30-day mortality %	R0 %	5-year survival %	10-year survival %
Sano et al. (2018)*	2001–2010	1539	13	1	< 1	25	1.5	90	41	28
Adam et al. (2006)*	1983–2004	1452	2	10	9	15	2.3	83	36	23
Groeschl et al. (2012)*	1990–2009	420	0	7	23	20	1.9	81	31	NR
Schiergens et al. (2016)	2003–2013	167	5	5	10	25	5	86	43	31
Hoffmann et al. (2015)	2001–2012	150	7	10	6	26	0.7	80	42	27
Takemura et al. (2013)	1993–2009	145	8	< 1	6	18	1.4	93	41	NR
Bohlok et al. (2020)	2005–2017	114	0	4	10	11	0	NR	56	27
Holzner et al. (2018)	1999–2015	100	12	14	6	NR	NR	90	57	34
Yedibela et al. (2005)	1978- 2001	162	0	3	5	26	4	62	26	NR
O'rourke et al. (2008)*	1986–2006	102	18	20	3	21	0.8	83	39	NR
Lendoire et al. (2007)*	1989–2006	106	0	6	22	NR	1.8	90	19	NR
Slotta et al. (2014)	NR	101	0	7	1	13	1	NR	30	NR
Present study*	1995–2018	637	3	5	5	NR	1.4	38	18	9
Present study R0-resected*	1995–2018	241	4	6	5	21	1.7	100	33	19

*means multicentric study

We studied surgically treated cases of non-colorectal non-neuroendocrine liver metastases from all hospitals in a well-defined geographical region. As expected, there were statistically significant differences between small and large hospitals with respect to case mix and treatment as well as in 5- and 10-year survival rates for all cases in the univariate analyses. In the multivariate analyses, however, we observed no statistically significant influence of the hospital category on 5- and 10-year survival rates.

5- and 10-year rates for observed survival were 18% and 9% for all cases, respectively, and 33% and 19%, respectively for R0-resected cases. These survival rates are similar to those in non-small cell lung carcinoma (Lu et al. 2019). 5- and 10-year rates for observed survival in the literature vary from 19 to 57% and 23% to 31%, respectively (Table 7). These differences are mainly due to case mix in the studies. Patients with breast cancer were included in 5% (Sano et al. (2018) to 61% (Bohlok et al. (2020), but most studies do not provide detailed information on tumor sites. Some studies combine primaries of ovary, kidney, urinary bladder, ureter, testis, and prostate to “genitourinary cancer” (Bohlok et al. 2020; Lendoire et al. 2007; Schiergens et al. 2016; Takemura et al. 2013; Yedibela et al. 2005). These groups include cancers with different prognosis. In our study, there is a statistically significant difference in survival between urinary bladder, ureter, and renal pelvis versus ovary (*p* = 0.012) and versus kidney (*p* = 0.017). We saw no primaries of the testis because metastases from these tumors are mainly treated with systemic therapy.

The median survival time of only 13 months for R0-resected patients with pancreatic adenocarcinoma is in agreement with other studies (Andreou et al. 2018; Hackert et al. 2017; Tachezy et al. 2016).

The percentage of GIST with excellent prognoses varies in studies with more than 100 patients from 0 to 18%, the percentage of sarcoma with good prognoses from < 1 to 23%. Since 2005, all GISTs are primarily treated by protein kinase inhibitors. Surgery is only indicated when systemic therapy fails.

In addition to differences in primary tumor sites and histological types, the lowest R0-rate is 62%, the highest 93%. In our study, residual tumor after liver resection was the most important negative factor for 5-year survival in all patients and in the subgroups of adenocarcinoma, kidney cancers, and breast cancers.

A short interval between diagnosis of the primary tumor and liver metastases also resulted independently in poorer 5-year survival. We saw in 65% of patients a tumor free interval of less than 24 months, others saw 50% or 51% (Bohlok et al. 2020; Holzner et al. 2018; Slotta et al. 2014).

Multiple liver metastases were the third factor having a negative impact on 5-year survival. In our study, 41% of patients had solitary liver metastases; in the literature, the percentage varies between 46 and 62% (Bohlok et al. 2020; Hoffmann et al. 2015; Lendoire et al. 2007; Sano et al. 2018; Takemura et al. 2013; Yedibela et al. 2005).

Male sex was a negative prognostic factor in all patients and in adenocarcinoma. Only in R0-resected cases influenced positive lymph nodes of the primary tumor the 5-year survival independently and statistically significant.

R-classification was in several studies (Adam et al. 2006; Groeschl et al. 2012; Holzner et al. 2018; Lendoire et al. 2007; Lucchese et al. 2018; Schiergens et al. 2016; Weitz et al. 2005) found to be an independent statistically significant factor for overall survival, as well as disease-free survival (Groeschl et al. 2012; Hoffmann et al. 2015; Holzner et al. 2018; Lendoire et al. 2007; Sano et al. 2018; Schmelzle et al. 2010; Weitz et al. 2005). In two studies the number of liver metastases was an independent statistically significant factor for overall survival (Sano et al. 2018; Schiergens et al. 2016).

In contrast to other studies, we did not find an independent statistically significant impact of extrahepatic tumor (Adam et al. 2006; Lucchese et al. 2018; O'rourke et al. 2008; Sano et al. 2018; Schiergens et al. 2016), lymphatic vessel invasion (Groeschl et al. 2012), type of liver resections (Adam et al. 2006), or diameter of metastases (Groeschl et al. 2012; O'rourke et al. 2008) on 5-year survival.

For breast cancer one needs to consider tumor biology, such as expression of progesterone and estrogen, HER2 receptor status and systemic treatment with hormone therapy, chemotherapy, and treatment with monoclonal antibodies to influence the prognosis in the metastatic stage markedly (Dittmar et al. 2013). In the present study, we saw for all 117 patients with breast cancer 5- and 10-year survival rates of 29% and 18%, respectively, for 64 radically resected patients (R0/R1) 5- and 10-year overall survival rates were 46% and 29%, respectively. These rates are similar to results in colorectal liver metastases. Yet even studies with control groups reach unequivocal conclusions on the benefit of resecting liver metastases from breast cancer (Millen et al. 2021; Sadot et al. 2016).

Similar results to breast cancer were achieved in radically resected ovarian cancer liver metastases with 5- and 10-year survival rates of 41% and 21%, respectively, and kidney cancer liver metastases with 5- and 10-year overall survival rates of 39% and 18%, respectively. These results are confirmed by others (Bauschke et al. 2021; Zhuo et al. 2020).

Most studies on liver resection in gastric carcinoma include Chinese or Japanese patients. In series with more than 20 resected patients, 5-year overall survival rates vary between 10 and 40% (Kataoka et al. 2017). We found 5- and 10-year overall survival rates of 17% and 7%, respectively for radically resected liver metastases of gastric adenocarcinoma. In a recently published paper, Jagric and Horvat (2020) found that synchronous liver resection in gastric cancer patients is safe and offers significant survival benefit compared to chemotherapy alone. Patients might expect similar long-term survival as patients with stages III and IV gastric cancer without liver metastases and R0 resection (Jagric and Horvat 2020).

For all other primaries in our study, sample sizes were too small to draw reliable conclusions by use of survival analysis. To overcome this problem, Adam et al. (2006) published a risk score for non-colorectal non-neuroendocrine liver metastases of mixed primaries which differentiates three groups (low, intermediate, and high risk) dependent on six factors in 2006. In our study as well as in others (Hoffmann et al. 2015; Lendoire et al. 2007; Sano et al. 2018), this score led to pair-wise statistically different prognostic groups. In our study, there were no patients with high-risk breast carcinoma. Moreover, in the multivariate analysis for breast cancer, only the R-classification had an independent statistically significant impact on 5-year survival.

## Conclusion

In studies investigating mixed primaries and different histological tumor entities, the most important factor for long-term survival is the case mix. We saw that complete resection of all tumor lesions at the time of liver resection leads to significantly better survival than incomplete procedures in most primaries. For R0-resected patients, we saw 5- and 10-year survival rates of 33% and 19%, respectively. In cases of bilobar metastases, a combination of surgical resection and ablation leads to similar results.

The Adam score (Adam et al. 2006) identifies some risk factors which influence prognosis in most but not in all tumor entities. For kidney cancer and breast cancer it can be simplified.

As long as it is impossible to initiate studies with uniform inclusion criteria and sample sizes similar to those published for colorectal cancer metastases the value of liver resection for patients with non-colorectal non-neuroendocrine liver metastases will remain an issue under discussion.
